# Robust Procedures for Estimating and Testing in the Framework of Divergence Measures

**DOI:** 10.3390/e23040430

**Published:** 2021-04-06

**Authors:** Leandro Pardo, Nirian Martín

**Affiliations:** 1 Department of Statistics and O. R., Faculty of Mathematics, Universidad Complutense de Madrid, 28040 Madrid, Spain; 2 Interdisciplinary Mathematics Institute, Complutense University of Madrid, 28040 Madrid, Spain; nirian@estad.ucm.es; 3 Department of Financial and Actuarial Economics & Statistics, Faculty of Commerce and Tourism, Complutense University of Madrid, 28003 Madrid, Spain

The approach for estimating and testing based on divergence measures has become, in the last 30 years, a very popular technique not only in the field of statistics, but also in other areas, such as machine learning, pattern recognition, etc. In relation to the estimation problem, it is necessary to minimize a suitable divergence measure between the data and the model under consideration. Some interesting examples of those estimators are the minimum phi-divergence estimators (MPHIE), in particular, these minimum Hellinger distance (MHD) and the minimum density power divergence estimators (MDPDE). The MPHIE (Pardo [1], Morales et al. [2]) are characterized by asymptotic efficiency (BAN estimators), the MHE (Beran [3]) by asymptotic efficiency and robustness inside the family of the MPHIE, and the MDPDE (Basu et al. [4]) by their robustness without a significant loss of efficiency as well as by the simplicity of getting them, because it is not necessary to use a nonparametric estimator of the true density function.

Based on these estimators of minimum divergence or distance, many people have studied the possibility to use them to obtain statistics for testing hypotheses. There are some possibilities to use them with that objective: (i) plugging them in a divergence measure in order to obtain the estimated distance (divergence) between the model, whose parameters have been estimated under the null hypothesis and the model evaluated in all of the parameter space, see, for instance, Martín and Pardo [5], Menéndez et al. [6], Salicrú et al. [7], Morales et al. [8,9]; (ii) extending the concept of the Wald test in the sense of considering MDPDE instead of maximum likelihood estimators (MLE). These test statistics have been considered in many different statistical problems: censoring, equality of means in normal and lognormal models, logistic regression model, multinomial regression in particular, and GLM models in general, etc., see, for instance, Basu et al. [10,11,12,13,14], Ghosh et al. [15], Castilla et al. [16], Ghosh et al. [17], and references therein; and, (iii) extending the concept of the Rao’s test in the sense of considering MDPDE instead of MLE, see Basu et al. [18] and Martín [19].

This Special Issue present new and original research papers that are based on MPHIE, MHD, and MDPDE, as well as test statistics that are based on these estimators from a theoretical and applied point of view in different statistical problems with special emphasis on robustness. Manuscripts give solutions to different statistical problems as model selection criteria based on divergence measures or in statistics for high-dimensional data with divergence measures as loss function are presented. It comprises nine selected papers that address novel issues, as well as specific topics illustrating the importance of the divergence measures or pseudodistances in statistics. In the following, the manuscripts are presented:

An important class of time-dynamic models is given by discrete-time integer-valued branching processes, in particular (Bienaymé-) Galton-Watson processes without immigration (GW), respectively, with immigration (GWI), which have numerous applications in biotechnology, population genetics, internet traffic research, clinical trials, asset price modelling, derivative pricing, and many others. As far as important terminology is concerned, they shall subsume both models as GW(I) and, simply as GWI in the case that GW appears as a parameter-special-case of GWI; recall that a GW(I) is called subcritical, respectively, critical, respectively, supercritical if its offspring mean is less than 1, respectively, equal to 1, respectively, larger than 1.

In “Some dissimilarity Measures of Branching Processes and optimal Decision Making in the Presence of Potential Pandemics”, Kammerer and Stummer, [20], compute exact values respectively bounds of dissimilarity/distinguishability measures—in the sense of the Kullback-Leibler information distance (relative entropy) and some transforms of more general power divergences and Rényi divergences—between two competing discrete-time Galton-Watson branching processes with immigration for which the offspring and the immigration (importation) are arbitrarily Poisson-distributed; especially, they allow for an arbitrary type of extinction-concerning criticality and, thus, for non-stationarity. They apply this to optimal decision making in the context of the spread of potentially pandemic infectious diseases (such as, e.g., the current COVID-19 pandemic), e.g., covering different levels of dangerousness and different kinds of intervention/mitigation strategies. Asymptotic distinguishability behavior and diffusion limits are also investigated by them. In a more concrete way, this paper pursues the following main goals:

(A) for any time horizon and any criticality scenario (allowing for non-stationarities), to compute lower and upper bounds—and sometimes even exact values—of the Hellinger integrals $H_{\lambda }(P_{A}\left|\right|P_{H})$, density power divergences $I_{\lambda }(P_{A}\left|\right|P_{H})$, and Rényi divergences $R_{\lambda }(P_{A}\left|\right|P_{H})$ of two alternative Galton-Watson branching processes $P_{A}$ and $P_{H}$ (on path/scenario space), where (i) $P_{A}$ has Poisson $\left(\beta_{A}\right)$ distributed offspring as well as Poisson $\left(\alpha_{A}\right)$ distributed immigration, and (ii) $P_{H}$ has Poisson $\left(\beta_{H}\right)$ distributed offspring as well as Poisson $\left(\alpha_{H}\right)$ distributed immigration; the non-immigration cases are covered as $\alpha_{A}=\alpha_{H}=0$; as a side effect, they also aim for corresponding asymptotic distinguishability results;
(B) to compute the corresponding limit quantities for the context in which (a proper rescaling of) the two alternative Galton-Watson processes with immigration converge to Feller-type branching diffusion processes, as the time-lags between the generation-size observations tend to zero; and,
(C) as an exemplary field of application, to indicate how to use the results that are pointed out in A) for Bayesian decision making in the epidemiological context of an infectious-disease pandemic (e.g., the current COVID-19), where e.g., potential state-budgetary losses can be controlled by alternative public policies (such as e.g., different degrees of lookdown) for mitigations of the time-evolution of the number of infectious persons (being quantified by a GW(I)). Corresponding Neyman-Pearson testing will also be treated.

Because of the involved Poisson distributions, these goals can be tackled with a high degree of tractability, which is worked out in detail with the following structure they first introduce (i) the basic ingredients of Galton-Watson processes, together with their interpretations in the above-mentioned pandemic setup, where it is essential to study all types of criticality (being connected with levels of reproduction numbers), (ii) the employed fundamental information measures, such as Hellinger integrals, power divergences, and Rényi divergences, (iii) the underlying decision-making framework, as well as (iv) connections to time series of counts and asymptotical distinguishability. Thereafter, they start other detailed technical analyses by giving recursive exact values respectively recursive bounds-as well as their applications-of Hellinger integrals $H_{\lambda }(P_{A}\left|\right|P_{H})$, density power divergences $I_{\lambda }(P_{A}\left|\right|P_{H})$, and Rényi divergences $R_{\lambda }(P_{A}\left|\right|P_{H})$. Explicit closed-form bounds of Hellinger integrals $H_{\lambda }(P_{A}\left|\right|P_{H})$ that will be worked are obtained as well as Hellinger integrals and power divergences of the above-mentioned Galton-Watson type diffusion approximations.

The change point problem is a core issue in time series analysis because changes can occur in underlying model parameters, owing to critical events or policy changes, and ignoring such changes can result in false conclusions. Numerous studies exist on change point analysis in time series models; refer to Kang and Lee, see [21] and Lee and Lee, see [22], and the articles cited therein, for the background and history of change points in integer-valued time series models. Lee and Lee [22], conducted a comparison study of the performance of various cumulative sum (CUSUM) tests while using score vectors and residuals through the Monte Carlo simulations. In their work, the conditional maximum likelihood estimator (CMLE) is used for the parameter estimation and the construction of the CUSUM tests. However, the CMLE is often damaged by outliers, and so is the performance of the CMLE-based CUSUM test. In general, outliers easily mislead the CUSUM test, since they can be mistakenly taken for abrupt changes; in the opposite, they can misidentify change points in their presence on time series.

In the work “Monitoring Parameter Change for Time Series. Models of Counts Based on Minimum Density Power Divergence estimator”, Lee and Kim [23] consider the CUSUM monitoring procedure to detect a parameter change for integer-valued generalized autoregressive heteroscedastic models (core area in time series analysis that includes diverse disciplines in social, physical, engineering, medical sciences, etc. Integer-valued autoregressive time series models and the integer-valued generalized autoregressive conditional heteroscedastic models have been widely studied in the literature and applied to various practical problems), whose conditional density of present observations over past information follows one parameter exponential family distributions. For this purpose, they use CUSUM of score functions that were deduced from the objective functions, constructed for the MDPDE that includes the MLE, to diminish the influence of outliers. It is well-known that, as compared to the MLE, the MDPDE is robust against outliers with little loss of efficiency. This robustness property is properly inherited by the proposed monitoring procedure. The CUSUM test has been a conventional tool to detect a structural change in underlying models, and it has been applied not only to retrospective change point tests, but also to on-line monitoring and statistical process control (SPC) problems, which were designed to monitor abnormal phenomena in manufacturing processes and health care surveillance. The CUSUM control chart has been popular due to its considerable competency in early detection of anomalies. A simulation study is conducted to affirm the validity of their method. Focus is placed on comparing the MDPDE-based CUSUM test with the MLE-based CUSUM test for Poisson INGARCH models to demonstrate the superiority of the former over the latter in the presence of outliers. A real data analysis of the return times of extreme events of Goldman Sachs Group (GS) stock prices is also provided to illustrate the validity of the proposed test. These authors, see [24], considered the CUSUM tests based on score vectors for the MLE and MDPDE in exponential family distribution INGARCH models.

In “Robust Change Point Test for General Integer-Valued Time Series Models Based on Density Power Divergence” by Kim and Lee [24], the problem of testing for a parameter change in general integer-valued time series models whose conditional distribution belongs to the one-parameter exponential family when the data are contaminated by outliers is considered. In particular, they use a robust change point test that is based on density power divergence (DPD) as the objective function of the MDPDE. The results show that, under regularity conditions, the limiting null distribution of the DPD-based test is a function of a Brownian bridge. Monte Carlo simulations are conducted to evaluate the performance of the proposed test and show that the test inherits the robust properties of the MDPDE and DPD. They compare the DPD-based test and the score-based CUSUM test to demonstrate the superiority of the proposed test in the presence of outliers. They provide a real data analysis of the return times of extreme events that are related to Goldman Sachs Group (GS) stock to illustrate the proposed tests.

MDPDE provides a general framework for robust statistics, depending on a parameter α, which determines the robustness properties of the method. The usual estimation method is numerical minimization of the power divergence. In “Robust Regression with Density Power Divergence: Theory, Comparisons, and Data Analysis”, by Riani et al. [25], is considered to be the special case of linear regression developing an alternative estimation procedure using the methods of S-estimation. The so obtained rho function is proportional to one minus a suitably scaled normal density raised to the power α. We used the theory of S-estimation to determine the asymptotic efficiency and breakdown point for this new form of S-estimation. Two sets of comparisons were made. In one, S power divergence is compared with other S-estimators using four distinct rho functions. The plots of efficiency against breakdown point show that the properties of S power divergence are close to those of Tukey’s biweight. The second set of comparisons is between S power divergence estimation and numerical minimization. Monitoring these two procedures in terms of breakdown point shows that the numerical minimization yields a procedure with larger robust residuals and a lower empirical breakdown point, thus providing an estimate of α, leading to more efficient parameter estimates.

Model selection is fundamental to the practical applications of statistics, and there is substantial literature on this issue. Classical model selection criteria include, among others, the Cp-criterion, the Akaike Information Criterion (AIC), based on the Kullback-Leibler divergence, and the Bayesian Information Criterion (BIC), as well as a General Information Criterion (GIC), which corresponds to a general class of criteria which also estimates the Kullback-Leibler divergence. These criteria have been proposed, respectively, in [26,27,28], and they represent powerful tools for choosing the best model among different candidate models that can be used to fit a given data set. On the other hand, many classical procedures for model selection are extremely sensitive to outliers and other departures from the distributional assumptions of the model. Robust versions of classical model selection criteria, which are not strongly affected by outliers, have been proposed, for example, in [29] and [30]. Some recent proposals for robust model selection are criteria based on divergences and minimum divergence estimators. Here, we recall the Divergence Information Criteria (DIC) based on the density power divergences that were introduced in [31], the Modified Divergence Information Criteria (MDIC) introduced in [32], and the criteria based on minimum dual divergence estimators introduced in [33]. In [34,35] some model selection criteria are presented. In “Robust Model Selection Criteria Based on Pseudodistances” by Toma et al. see [34], a new class of robust model selection criteria are introduced. These criteria are defined by estimators of the expected overall discrepancy using pseudodistances and the minimum pseudodistance principle. The theoretical properties of these criteria are proved, namely asymptotic unbiasedness, robustness, consistency, as well as the limit laws. The case of the linear regression models is studied and a specific pseudodistance based criterion is proposed. Monte Carlo simulations and applications for real data are presented to exemplify the performance of the new methodology. These examples show that the new selection criterion for regression models is a good competitor of some well known criteria and may have superior performance, especially in the case of small and contaminated samples.

Classical likelihood function requires the exact specification of the probability density function, but, in most applications, the true distribution is unknown. In some cases, where the data distribution is available in an analytic form, the likelihood function is still mathematically intractable due to the complexity of the probability density function. There are many alternatives to the classical likelihood function; one of them is the composite likelihood. Composite likelihood is an inference function that is derived by multiplying a collection of component likelihoods; the particular collection used is a conditional determined by the context. Therefore, the composite likelihood reduces the computational complexity, so that it is possible to deal with large datasets and very complex models, even when the use of standard likelihood methods is not feasible. Composite likelihood methods have been successfully used in many applications concerning, for example, genetics, generalized linear mixed models, spatial statistics, frailty models, multivariate survival analysis, etc. Asymptotic normality of the composite maximum likelihood estimator (CMLE) still holds with the Godambe information matrix to replace the expected information in the expression of the asymptotic variance-covariance matrix. This allows for the construction of composite likelihood ratio test statistics, Wald-type test statistics, as well as score-type statistics. Varin [36] provides a review of composite likelihood methods. They mentioned, at this point, that CMLE, as well as the respective test statistics are seriously affected by the presence of outliers in the set of available data. In this sense, [37,38,39] derived some new distance-based estimators and tests with good robustness behavior without an important loss of efficiency. In the context of the composite likelihood there are some criteria based on Kullback-Leibler divergence, see, for instance [40,41,42] and references therein. To the best of our knowledge, only Kullback-Leibler divergence was used to develop model selection criteria in a composite likelihood framework. To fill this gap, our interest is now focused on DPD. In “Model Selection in a Composite Likelihood Framework Based on Density Power Divergence”, Castilla et al. see [35], consider the composite minimum density power divergence estimator (CMDPDE), as introduced in [37], in order to present a model selection criterion in a composite likelihood framework. The criterion introduced in [37] will be called composite likelihood DIC criterion (CLDIC). The motivation, as pointed out by the authors, of considering a criterion based on DPD instead of Kullback-Leibler divergence is due to the robustness of the procedures based on DPD in statistical inference, not only in the context of full likelihood, but also in the context of composite likelihood [37,38]. After introducing the new model selection criterion, CLDIC, based on CMDPDE, some of its asymptotic properties are studied. A simulation study is carried out and some numerical examples are also presented.

Bounding the best achievable error probability for binary classification problems is relevant to many applications, including machine learning, signal processing, and information theory. The Bayes error rate is the expected risk for the Bayes classifier, which assigns a given feature vector x to the class with the highest posterior probability. The Bayes error rate is the lowest possible error rate of any classifier for a particular joint distribution. The Bayes error rate provides a measure of classification difficulty. Thus, when known, the Bayes error rate can be used to guide the user in the choice of classifier and tuning parameter selection. In practice, the Bayes error is rarely known and it must be estimated from data. The estimation of the Bayes error rate is difficult due to the non-smooth in function within an integral. Thus, research has focused on deriving tight bounds on the Bayes error rate based on smooth relaxations of the min function. Many of these bounds can be expressed in terms of divergence measures between the pair of class distributions, such as the Bhattacharyya distance or Jensen-Shannon divergence measure. Many techniques have been developed for estimating divergence measures. These methods can be broadly classified into two categories: (i) plug-in estimators in which we estimate the probability densities and then plug them in the divergence function and (ii) entropic graph approaches, in which the relationship between the divergence function and a graph functional in Euclidean space is derived. Examples of plug-in methods include k-nearest neighbor (K-NN) and Kernel density estimator (KDE) divergence estimators. Examples of entropic graph approaches include methods that are based on minimal spanning trees (MST), K-nearest neighbors graphs (K-NNG), minimal matching graphs (MMG), traveling salesman problem (TSP), and their power-weighted variants. Recently, the Henze-Penrose (HP) divergence has been proposed for bounding classification error probability. In “Convergence Rates for Empirical Estimation of Binary Classification Bounds”, by Sekeh et al. see [43], the problem of empirically estimating the HP-divergence from random samples is considered. The first contribution of this paper is that they obtain a bound on the convergence rates for the Friedman and Rafsky (FR) estimator of the HP-divergence, which is based on a multivariate extension of the non-parametric run length test of equality of distributions. This estimator is constructed using a multicolored MST on the labeled training set, where MST edges connecting samples with dichotomous labels are colored differently from edges connecting identically labeled samples. While previous works have investigated the FR test statistic in the context of estimating the HP-divergence, to the best of the author’s knowledge, its minimax MSE convergence rate has not been previously derived. The bound on convergence rate is established by using the umbrella theorem, for which they define a dual version of the multicolor MST. The proposed dual MST in this work is different than the standard dual MST that was introduced by Yukich in [44]. They show that the bias rate of the FR estimator is bounded by a function of *N*, η and *d*, as $O\left(N^{-\eta^{2}/\left(d\left(\eta +1\right)\right)}\right)$, where *N* is the total sample size, *d* is the dimension of the data samples d>2, and η is the Hölder smoothness parameter 0≤η≤1. They also obtain the variance rate bound as $O(N^{-1}).$ The second contribution of this paper is a new concentration bound for the FR test statistic. The bound is obtained by establishing a growth bound and a smoothness condition for the multicolored MST. Because the FR test statistic is not a Euclidean functional, we cannot use the standard subadditivity and superadditivity approaches. Their concentration inequality is derived using a different Hamming distance approach and a dual graph to the multicolored MST. They experimentally validate their theoretic results comparing the MSE theory and simulation in three experiments with various dimensions d = 2, 4, 8. They observe that, in all three experiments, as sample size increases, the MSE rate decreases and, for higher dimensions, the rate is slower. Our theory matches the experimental results in all sets of experiments.

In “Distance-Based Estimation Methods for Models for Discrete and Mixed-Scale Data” by Sofikitou et al. [45], robust methods for mixed-scale data are developed. Mixed-scale measurements scenario have both discrete (categorical or nominal) and continuous type random variables. Initially, they reviews basic concepts in minimum disparity estimation (MDE), which has been extensively studied in models where the scale of the data is either interval or ratio ([3,12]). It has also been studied in the discrete outcomes case. Specifically, when the response variable is discrete and the explanatory variables are continuous, Pardo et al. [46] introduced a general class of distance estimators based on ϕ-divergence measures, the MPHIE, and they studied their asymptotic properties. The estimators can be viewed as an extension/generalization of the MLE. In Pardo et al. [47], the MPHIE is used in statistic to perform goodness-of-fit tests in logistic regression models, while Pardo and Pardo [48] extended the previous works to address solving problems for testing in generalized linear models with binary scale data. The case where data are measured on discrete scale (either on ordinal or generally categorical scale) has also attracted the interest of other researchers. For instance, Simpson [49] demonstrated that minimum Hellinger distance estimators fulfill desirable robustness properties and, for this reason, can be effective in the analysis of count data that are prone to outliers. Simpson [50] also suggested tests based on the minimum Hellinger distance for parametric inference that are robust as the density of the (parametric) model can be nonparametrically estimated. In contrast, Markatou et al. [51] used weighted likelihood equations to obtain efficient and robust estimators in discrete probability models and applied their methods to logistic regression, whereas Basu and Basu [52] considered robust penalized minimum disparity estimators for multinomial models with good small sample efficiency. Moreover, Gupta et al. [53], Martín and Pardo [54], and Castilla et al. [55] used the MPHIE to provide a solution to testing problems in polytomous regression models. Working in a similar fashion, Martín and Pardo [56] studied the properties of the family of MPHIE for log-linear models with linear constraints under multinomial sampling to identify the potential associations between various variables in multi-way contingency tables. Pardo and Martín [57] presented an overview of works that are associated with contingency tables of symmetric structure on the basis of MPHIE and ϕ-divergence test statistics. Additional works include Pardo and Pardo [58] and Pardo et al. [59]. Basu et al. [60] introduced alternative power divergence measures. Afterwards, define various Pearson residuals appropriate for the measurement scale of the data and study their properties. They further concentrate on the case of mixed-scale data, which is, data measured in both categorical and interval scale. We study the asymptotic properties and the robustness of MDE obtained in the case of mixed-scale data and exemplify the performance of the methods via simulation. The results show that, depending on the level of contamination and the type of contaminating probability model, the performance of the methods is satisfactory.

The asymptotic distributions of minimum Hellinger distance estimators has been well investigated; nevertheless, the probabilities of rare events that are induced by them are largely unknown. In “Event Analysis for Minimum Hellinger Distance Estimators via Large Deviation Theory” by Vidayashankar and Collamore [61], rare event probabilities, for the minimum Hellinger distance estimators of a family of continuous distributions satisfying an equicontinuous condition, using large deviation theory under a potential model misspecification, in both one and higher dimensions are analyzed. They show that these probabilities decay exponentially, characterizing their decay via a “rate function”, which is expressed as a convex conjugate of a limiting cumulant generating function. In the analysis of the lower bound, in particular, certain geometric considerations arise, which facilitate an explicit representation, also in the case when the limiting generating function is non-differentiable. The analysis also involves the modulus of continuity properties of the affinity, which may be of independent interest. The results that are presented in this paper extend large deviation asymptotics for M-estimators that were given previously. In contrast to the case for M-estimators, our setting is complicated due to its inherent nonlinearity, leading to complications in the proofs of both the upper and lower bounds, and an unexpected subtlety in the form of the rate function for the lower bound. The results of Vidayashankar and Collamore (2021) suggest that one can, under additional hypotheses, establish saddlepoint approximations to the density of minimum Hellinger distance estimators, which would enable one to sharpen inference for small samples.

Similar results are expected to hold for discrete distributions. However, the equicontinuity condition is not required in that case, since $\ell_{1}$, unlike $L_{1}\left(S\right)$ (the space of integrable functions on *S*), possesses the Schur property. Hence, the large deviation principle in the weak topology of $\ell_{1}$ can be derived (more easily) using a standard Gartner-Ellis argument and, utilizing this, one can, in principle, repeat all of the arguments above to derive results that are analogous to Theorems 2.2 and 2.3. Large deviations for other divergences under weak family regularity (such as non-compactness of the parameter space) and their connections to estimation and test efficiency are interesting open problems that require new techniques beyond those that are described in this article.
